# The role of the prefrontal cortex in cocaine-induced noradrenaline release in the nucleus accumbens: a computational study

**DOI:** 10.1007/s00422-025-01005-5

**Published:** 2025-02-07

**Authors:** Samuele Carli, Aurelia Schirripa, Pierandrea Mirino, Adriano Capirchio, Daniele Caligiore

**Affiliations:** 1 Computational and Translational Neuroscience Laboratory, Institute of Cognitive Sciences and Technologies, National Research Council (CTNLab-ISTC-CNR), Via Gian Domenico Romagnosi, 18A, 00196 Rome, Italy; 2 Entersys s.r.l., Via San Pio X 44, 35027 Noventa Padovana, Padua, Italy; 3 AI2Life s.r.l., Innovative Start-Up, ISTC-CNR Spin-Off, Via Sebino 32, 00199 Rome, Italy

**Keywords:** Addiction, Cocaine, Locus coeruleus, Medial prefrontal cortex, Microdialysis, Motivation, Noradrenaline, Nucleus accumbens, Ordinary differential equations, System-level computational modeling

## Abstract

Research has extensively explored the role of the dopaminergic system in the reward circuit, while the contribution of the noradrenergic system remains less understood. This study aims to fill this gap by employing computational modeling to examine how the medial prefrontal cortex (mPFC) influences cocaine-induced norepinephrine (NE) release in the nucleus accumbens shell (NAcc), with mediation by the nucleus of the tractus solitarius (NTS) and the locus coeruleus (LC). The model replicates previously reported data on NE release in the mPFC following cocaine administration. Additionally, it predicts that NE depletion in the mPFC affects NE release in the NAcc through interactions with the NTS and LC. This work proposes a system-level hypothesis, suggesting that the mPFC regulates NE release in the NAcc by modulating the LC and NTS. These findings enhance our understanding of the neurochemical response to cocaine and offer potential directions for future addiction treatments.

## Introduction

All species with a basilar capacity for cognition seek reward and gratification. However, understanding the neural mechanisms behind this behavior is challenging due to the involvement of multiple brain regions and neurotransmitters and the complexity of their interactions. Unraveling these mechanisms could lead to more effective treatments for psychopathologies related to reward and punishment, such as depression and addiction (Aupperle and Paulus 2010; Eshel and Roiser 2010; Myers et al 2016). Over the years, research has strongly supported the role of dopamine (DA) in the reward system (Chaua et al 2018; Schultz 2002; de Jong et al 2022) and its involvement in the development of addictive disorders (Diana 2011; DiSegni et al 2020; Cassidy et al 2020; Samaha et al 2021).

However, researchers have paid less attention to noradrenaline (NE), another neurotransmitter that plays a significant role in addiction-related behaviors and is involved in reward and motivation systems (Puglisi-Allegra and Ventura 2012; Vanderschuren et al 2003; Brown et al 2009). NE, one of the most abundant neurotransmitters in the brain and peripheral tissues, is involved in various behavioral processes, including arousal, attention, appetite, and stress regulation (Aston-Jones and Cohen 2005; Berridge and Waterhouse 2003; Sara and Bouret 2012; Holland et al 2021). The central noradrenergic system consists of four nuclei: the rostroventrolateral medulla, the nucleus of the tractus solitarius (NTS), the locus coeruleus (LC), and the subcoeruleus. Projections from these areas innervate nearly every brain region (Moore and Bloom 1979; Robertson et al 2013). The LC, which contains the majority of NE-producing neurons (Aston-Jones and Waterhouse 2016; Poe et al 2020), projects to several brain regions, including the medial prefrontal cortex (mPFC) and the nucleus accumbens shell (NAcc) (Chandler et al 2013, 2014; Delfs et al 1998; Noei et al 2022).

The prefrontal-accumbal (mPFC-NAcc) circuit is critical for attributing salience to both aversive and rewarding stimuli (Ventura et al 2003, 2007). Selective NE depletion in the mPFC of mice impairs conditioned place preference (CPP) induced by amphetamine, cocaine, and morphine, as well as the reinstatement of extinguished morphine-induced CPP (Ventura et al 2003, 2006, 2007). Additionally, the injection of an $\alpha$1AR antagonist into the mPFC blocks the cocaine-induced reinstatement of cocaine-seeking behaviors (?).

Notably, prefrontal cortical NE transmission is essential for attributing motivational salience to both reward- and aversion-related stimuli by modulating DA in the NAcc, a brain area involved in all motivated behaviors (Ventura et al 2003, 2006, 2007). Substances of abuse also influence NE transmission in the NAcc. McKittrick and Abercrombie (2007) demonstrated increased NE release in the NAcc following amphetamine administration. In addition to the mPFC and NAcc, the LC projects to the NTS (Delfs et al 1998). The NTS role in autonomic function is well established (Balaban and Beryozkin 1994; Clark et al 2011), while few studies have explored its involvement in reward-related processes. Interestingly, rescuing NE deficits in the NTS- but not in the LC-restores morphine-induced CPP (Olson et al 2006), and projections from the NTS to the amygdala are crucial for morphine-associated memory destabilization (Zheng et al 2022).

This study investigates for the first time an extended circuit that explores the role of the mPFC in modulating cocaine-induced NE release in the NAcc through the NTS and LC. Previous research has shown that prefrontal NE depletion can abolish cocaine-induced NE release in the mPFC and DA release in the NAcc, as well as cocaine-induced CPP (Ventura et al 2007). This paper posits that NE depletion in the mPFC could also impact NE release in the NAcc, potentially mediated by the circuit involving NE transmission in the NTS and LC. The article proposes a computational model based on ordinary differential equations to test this hypothesis. In particular, the model replicates previously reported results on the effects of cocaine on NE release in the mPFC (Devoto et al 2014; Florin et al 1994; Ventura et al 2007). It successfully reproduces these data, highlighting for the first time the critical role of the NTS in modulating NAcc NE release. A stability analysis assesses the robustness of the mathematical formulation used in designing the model. This analysis represents a crucial step in validating the model effectiveness.

The simulation results suggest that the mPFC can effectively influence cocaine-induced NE release in the NAcc through modulation of the NTS and LC. These findings generate testable predictions for new in vivo microdialysis experiments. Such experiments should verify that cocaine increases NE outflow in both the mPFC and NAcc, and that NE depletion in the prefrontal cortex reduces cocaine-induced NE release in the NAcc. Thus, the model supports a system-level hypothesis supporting the mPFC modulatory role in accumbal NE release through its influence on the LC and NTS. This knowledge contributes to a deeper understanding of the brain NE response to cocaine.

## Methods

### Model architecture

**Fig. 1:**
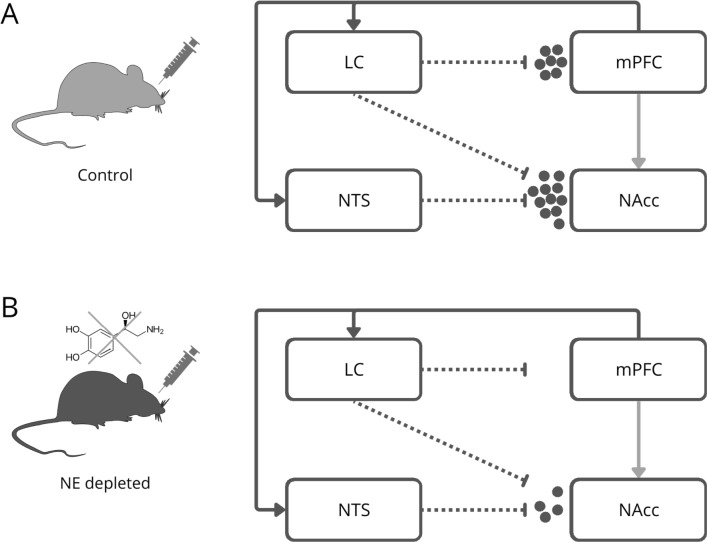
Model architecture illustrating the locus coeruleus (LC), medial prefrontal cortex (mPFC), nucleus of the tractus solitarius (NTS), and nucleus accumbens shell (NAcc). Excitatory connections between the mPFC and both the LC and NTS are depicted with arrows. Dashed lines represent norepinephrine (NE) release in the NAcc from the LC and NTS. Cocaine-induced NE release in the NAcc of control mice (**A**). Cocaine-induced NE release in the NAcc of mPFC NE-depleted mice (**B**)

The model architecture reproduces the dynamical interaction between four brain areas (Fig. 1): LC, mPFC, NTS, NAcc. To simplify the NE circuit, we isolated the two most important NEergic sources (Poe et al 2020; Bundzikova-Osacka et al 2015), and the two principal targets implicated in the reward process (Piantadosi et al 2021). The connections from mPFC to LC and NTS are mostly excitatory as reported in literature (Jodo et al 1998; Eden and Buijs 2000; Owens et al 1999). NE projections from LC to mPFC are also reported in literature (Florin-Lechner et al 1996; Tomassini et al 2022). Data relative to cocaine-induce NE increase in mPFC are taken from Ventura et al (2007). NE projections from LC and NTS to NAcc Shell are reported in literature, in particular NTS has a major projection in Shell (Delfs et al 1998; Holt 2022).

The mPFC projects directly to both the NAcc core and shell, with distinct functional implications for reward processing and addiction-related behaviors. Specifically, the NAcc core is often implicated in the initiation of reward-related behaviors, while the shell plays a more prominent role in processing motivation and affective responses associated with drug use (Piantadosi et al 2020, 2021; Delfs et al 1998). However, in the current model, the focus was placed primarily on the indirect pathway through the LC and NTS due to the model scope, which aims to isolate the influence of the prefrontal cortex on the noradrenergic system response to cocaine, with particular emphasis on the dynamic interactions within the NAcc shell. The exclusion of direct mPFC to NAcc projections was made to simplify the model and avoid overcomplicating the network, thereby enabling a clearer investigation of the mPFC regulatory influence through the LC and NTS. Nevertheless, the contribution of mPFC projections to the NAcc core should not be underestimated. These direct projections could certainly modulate the neural dynamics observed in both the core and shell regions, possibly influencing the overall reward-related effects of cocaine. Future versions of the model could incorporate these projections to further explore how mPFC signaling differentially affects the NAcc core and shell during cocaine administration.

### Model equations

The following differential equations system simulates the activity of the model components and their dynamical interactions:

1 $$\begin{aligned} {\left\{ \begin{array}{ll} \dot{\textrm{LC}} = -\tau _{\textrm{LC}} \textrm{LC} + \alpha ^{\textrm{mPFC}}_{\textrm{LC}} \textrm{mPFC} + \alpha ^{\textrm{ext}}_{\textrm{LC}}\\ \dot{\textrm{NE}_\textrm{mPFC}} = -l k(t) \tau ^{\textrm{NE}}_{\textrm{mPFC}} {\textrm{NE}_{\textrm{mPFC}} + l \alpha ^{\textrm{LC}}_{\textrm{mPFC}} \textrm{LC}}\\ \dot{\textrm{mPFC}} = -\tau _{\textrm{mPFC}}{\textrm{mPFC} + \alpha ^{\textrm{NE}}_{\textrm{mPFC}} {\textrm{NE}_{\textrm{mPFC}}} + \alpha ^{\textrm{ext}}_{\textrm{mPFC}}}\\ \dot{\textrm{NTS}} = -\tau _{\textrm{NTS}} {\textrm{NTS} + \alpha ^{\textrm{mPFC}}_{\textrm{NTS}} \textrm{mPFC} + \alpha ^{\textrm{ext}}_{\textrm{NTS}}}\\ \dot{\textrm{NE}_\textrm{NAcc}} = -k(t) \tau ^{\textrm{NE}}_{\textrm{NAcc}} {\textrm{NE}_{\textrm{NAcc}} + \alpha ^{\textrm{LC}}_{\textrm{NAcc}} \textrm{LC} +\alpha ^{\textrm{NTS}}_{\textrm{NAcc}} \textrm{NTS}}\\ \dot{\textrm{NAcc}} = -\tau _{\textrm{NAcc}} \textrm{NAcc} + \alpha ^{\textrm{NE}}_{\textrm{NAcc}} {\textrm{NE}_{\textrm{NAcc}} + \alpha ^{\textrm{ext}}_{\textrm{NAcc}}} \\ \end{array}\right. } \end{aligned}$$

Depletion was simulated by changing $l$ from 1 in Sham to 0 in NE-depleted virtual mice. This statement refers to a specific condition applied during simulations of NE depletion. By setting the derivative $\dot{\textrm{NE}}_{\textrm{mPFC}} = 0$, the model effectively maintains a constant level of $\textrm{NE}_{\textrm{mPFC}}$ throughout the simulation. This approach simulates a scenario where the mPFC experiences NE depletion, preventing it from responding to external stimuli, such as cocaine. While this simplification allows the model to represent the effect of NE depletion, it does not fully capture the dynamic process of NE reduction over time. A more biologically accurate model would simulate a gradual decrease in NE levels, rather than maintaining a constant value, reflecting the depletion of NE from synaptic reserves and its reduced availability. When this condition is undone (i.e., when the system is allowed to evolve dynamically), the equation for $\textrm{NE}_{\textrm{mPFC}}$ becomes active again, allowing the model to reflect the normal interactions and fluctuations of NE in response to various stimuli. This dynamic is crucial for understanding how the mPFC responds under different conditions, including both sham and depleted states. The contribution of this differential equation lies in its ability to model the impact of NE on mPFC functionality. By incorporating this equation, the model captures the essential role of NE in modulating mPFC activity, which is particularly important when investigating the effects of substances like cocaine that influence neurotransmitter systems.

The cocaine effect, starting at time $t_c$, was simulated using a kernel function $k(t)$ defined as:

2 $$\begin{aligned} {\left\{ \begin{array}{ll} k(t) = 1, \quad t \le t_c \\ k(t) = 1 - a ( e^{-\frac{(t-t_c)^2}{b}} - e^{-\frac{(t-t_c)^2}{c}} ), \quad t > t_c \end{array}\right. } \end{aligned}$$

**Fig. 2:**
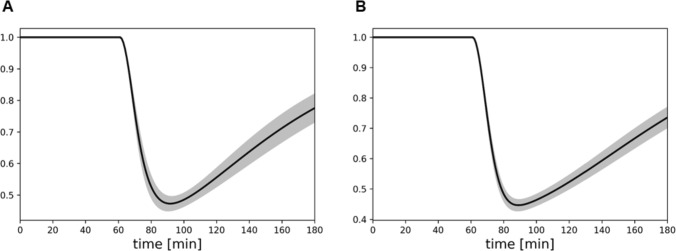
Shape of the kernel function in both Sham mice (**A**) and NE-depleted mice (**B**). The parameters $a$, $b$, and $c$ of Eq. (2) used to generate these graphs were fitted using a genetic algorithm

The kernel function $k(t)$, used to model the effects of cocaine, captures the temporal dynamics of its influence on NE levels. The parameters of the kernel function $a$, $b$, and $c$ are selected to ensure that $k(t)$ remains within the physiological bounds $[0,1]$, representing realistic neurotransmitter levels. The significance of these parameters is discussed in detail in subsequent sections (Tables 1 and 2). Cocaine exhibits a gradual onset of action, starting at time $t_c$, and its effect is represented by a monotonically increasing function that modulates NE levels and describes their return to basal states over time.

Cocaine acts as a psychostimulant, producing various behavioral and molecular effects (Johanson and Fischman 1989; Bravo et al 2022). Its pharmacokinetics are complex, primarily due to its interaction with multiple neurotransmitter systems, including the blockade of dopamine (DAT), serotonin (SERT), and norepinephrine (NET) transporters (Kuhar et al 1991; Johanson and Fischman 1989; Bravo et al 2022). This blockade elevates neurotransmitter concentrations in the synaptic cleft, resulting in a range of behavioral and physiological responses. Foundational studies have shown that cocaine action on these transporters is critical for understanding its psychostimulant effects and its role in addiction (Chao and Nestler 2004; Nestler 2005).

Previous research has explored the complexities of cocaine effects using various computational approaches. Modeling its pharmacokinetics is inherently difficult, with only a few papers addressing this challenge (Pendyam et al 2009; Zheng and Zhan 2012). For example, Pan et al (1991) developed a mathematical model of cocaine metabolism in the brain and body, illustrating the intricate dynamics involved in its pharmacological effects. To simplify the modeling of cocaine complex mechanisms, which involve not only neurotransmitters but also enzymatic processes, this study focuses specifically on NE modulation. A monotonic increasing kernel function models the effect of cocaine, simplifying its dynamics by focusing on the essential features of NE regulation. This approach isolates the influence of cocaine on NE, facilitating a more tractable analysis of its behavioral and molecular impacts.

This study employs a mean-field model to simulate neural dynamics. Mean-field models are widely used in computational neuroscience to simplify large-scale neural networks by averaging the activity of individual neurons into a collective dynamic. This approach effectively captures the macroscopic behavior of neural circuits while minimizing computational complexity. The mean-field approximation models emergent behaviors in neural populations without tracking the detailed activity of each neuron. By averaging over large populations, the focus shifts to the overall trends and patterns arising from network interactions. This method has been successfully applied in various contexts to model both spontaneous and task-related brain activity, as well as its modulation by external inputs and neuromodulators (Deco et al 2008; Muller et al 2016; La Camera 2021; Alexandersen et al 2024).

### Simulation settings

The model was developed using the Python programming language. The code implementing the model, the genetic algorithm used to find the free parameters, and the values of the free parameters fitted by the genetic algorithm are available here: https://github.com/ctnlab/NTS_NE/

#### Tuning the model parameters

Table 1 summarizes the biological meaning of free parameters. The values of the model parameters were set to obtain a steady-state for the variables of the equation similar to those found in experimental observations (see Table 2). In particular, the parameter settings were optimized through an automatic procedure known as a “genetic algorithm”. This method mimics the process of natural selection, where candidate solutions (parameter sets) are iteratively evolved to improve their performance. The algorithm begins by generating an initial population of random parameter sets. Each set is evaluated based on a fitness function, which in this case measures the accuracy of the model in reproducing the experimental data. The best-performing sets are selected, combined, and mutated to produce a new generation of parameter sets. This process repeats until the algorithm converges on an optimal or near-optimal solution. The reader can find more details on this procedure in Caligiore et al (2017, 2021). The free parameters determined by the genetic algorithm, along with the steady-state activity values reported in Table 2, support the unique implementation of each brain region (NTS, NAcc, mPFC, LC) in the model.

**Table 1:** Free parameters of the equations fitted by the genetic algorithm

Parameter	Meaning	Unit
$\tau _{\text {LC}}$	Decay constant of LC neurons	Hz
$\alpha _{\text {LC}}^{\text {mPFC}}$	excitation of mPFC on LC	Hz
$\alpha _{\text {LC}}^{\text {ext}}$	External drive to LC	Hz/s
$\tau _{\text {mPFC}}^{\text {NE}}$	Decay constant of NE in mPFC	Hz
$\alpha _{\text {mPFC}}^{\text {LC}}$	Influence of NE release on mPFC	pg
$\tau _{\text {mPFC}}$	Decay constant of mPFC neurons	Hz
$\alpha _{\text {mPFC}}^{\text {NE}}$	Excitation of NE on mPFC	(Hz /s) $\cdot$ (1 /pg)
$\alpha ^{\text {ext}}_{\text {mPFC}}$	External drive to mPFC	Hz/s
$\tau _{\text {NTS}}$	Decay constant of NTS neurons	Hz
$\alpha _{\text {NTS}}^{\text {mPFC}}$	Excitation of mPFC on NTS	Hz
$\alpha _{\text {NTS}}^{\text {ext}}$	External drive to NTS	Hz/s
$\tau _{\text {NAcc}}^{\text {NE}}$	Decay constant of NE in NAcc	Hz
$\alpha _{\text {NAcc}}^{\text {LC}}$	Influence of NE release on NAcc (LC)	pg
$\alpha _{\text {Nacc}}^{\text {NTS}}$	Influence of NE release on NAcc(NTS)	pg
$\tau _{\text {NAcc}}$	Decay constant of NAcc neurons	Hz
$\alpha _{\text {NAcc}}^{\text {NE}}$	Excitation of NE on NAcc	(Hz /s) $\cdot$ (1 /pg)
$\alpha _{\text {NAcc}}^{\text {ext}}$	External drive to NAcc	Hz / s

The values of these parameters (170 values in total) can be reproduced by running the code available in the repository at the provided link (Sec. 2.3)

**Table 2:** Average activity at steady state for the variables in the equations of the system Eq. (1)

Parameters	Activity	Refrence
LC	2.3 Hz	(Szabo and Blier 2001)
$\text {NE}_{\text {mPFC}}$	0.4 pg	(DiSegni et al 2016)
mPFC	2.05 Hz	(Adhikari et al 2011)
NTS	1.85 Hz	(Ezure and Tanaka 2000; Robertson et al 2013)
NAcc	1.55 Hz	(Brady 2005)
$\text {NE}_{\text {NAcc}}$	0.4 pg	(DiSegni et al 2016)

#### Simulation configuration

The model equations are numerically integrated with a time step of $\Delta t = 1$, where each simulation step corresponds to $1~\text {min}$ of real-time. The simulation runs for a total of $180~\text {min}$. In line with in vivo microdialysis experiments, the mean of three samples is collected before cocaine administration at $60~\text {min}$, and subsequent samples are taken every $20~\text {min}$.

Data were collected from 10 different simulated mice, each generated by running the model with distinct seeds from a pseudo-random number generator. This resulted in variations in the noise signal affecting the model. Consequently, fewer mice may be required for in vivo replication, in line with the 3Rs principle (Replacement, Reduction, Refinement). In this study, individual variability was accounted for by using different seeds in the simulations. While this approach is simplistic, it is commonly used in computational neuroscience. By utilizing different seeds, the model parameters vary with each instantiation, thereby simulating individual differences across subjects. This method is useful for examining the impact of variability and capturing a broader range of potential outcomes in the simulations. The use of random seeds to model individual variability has been discussed in several studies (Dayan and Abbott 2005; Anastasio 2010; Caligiore et al 2014; Caligiore and Mirino 2020).

This study acknowledges the challenge posed by the mismatch in timescales between the fast neural dynamics inherent in conventional mean-field models and the longer timescales relevant to the 180-minute experimental simulation. Neural systems inherently exhibit a separation of timescales, where faster dynamics, such as spiking and synaptic transmission, occur on the order of milliseconds to seconds, while slower processes like neuromodulation, synaptic plasticity, and metabolic regulation influence behavior over minutes to hours. To address this challenge, the model adopts a simplified framework that leverages this timescale separation. Specifically, it treats the fast dynamics as quasi-steady-state processes, where rapid fluctuations stabilize over short-duration windows. These windows then get modulated by slower variables, such as neuromodulatory influences, which act over longer timescales. This approach enables efficient behavior modeling over extended periods without losing the essential dynamics of the faster processes. Previous studies (Rolls et al 2008; Pfeffer et al 2021; Kringelbach et al 2020) document the role of neuromodulation on slower neural processes. The model incorporates these insights to reproduce neuromodulatory effects within the simulation framework and to capture the interaction between fast and slow dynamics more realistically. By integrating this multi-timescale approach, the model more accurately approximates the neurophysiological processes underlying behavior during extended experiments while simplifying the computational demands of simulating fast dynamics over long timescales. This strategy aligns with empirical findings on neuromodulatory control over slower neural processes and enhances the interpretability of simulation outcomes over the 180-minute timescale.

### Statistical analysis

Differences between activities in the brain regions were analyzed using a Student’s t-test (with Welch’s correction when applicable) or the Mann–Whitney test. Normality of distribution was assessed with the Shapiro-Wilk test and the D’Agostino & Pearson test. For NE release in the mPFC and NAcc, percent changes from baseline levels were evaluated using repeated measures ANOVA, with Sidak’s multiple comparisons test applied where appropriate. When data were non-normally distributed, the Mann–Whitney test was used.

## Results

This section presents the main outcomes of the simulations, examining the effects of cocaine administration and NE depletion on neural activity and NE release in key brain regions. The first analysis focuses on the changes in firing activity across different brain regions during simulated cocaine injection. The following analysis investigates how NE depletion affects cocaine-induced NE release in the mPFC and NAcc. Finally, the stability of the model is assessed to confirm the robustness of the results under varying parameter conditions.

### Alterations in brain region activity following simulated cocaine administration

**Fig. 3:**
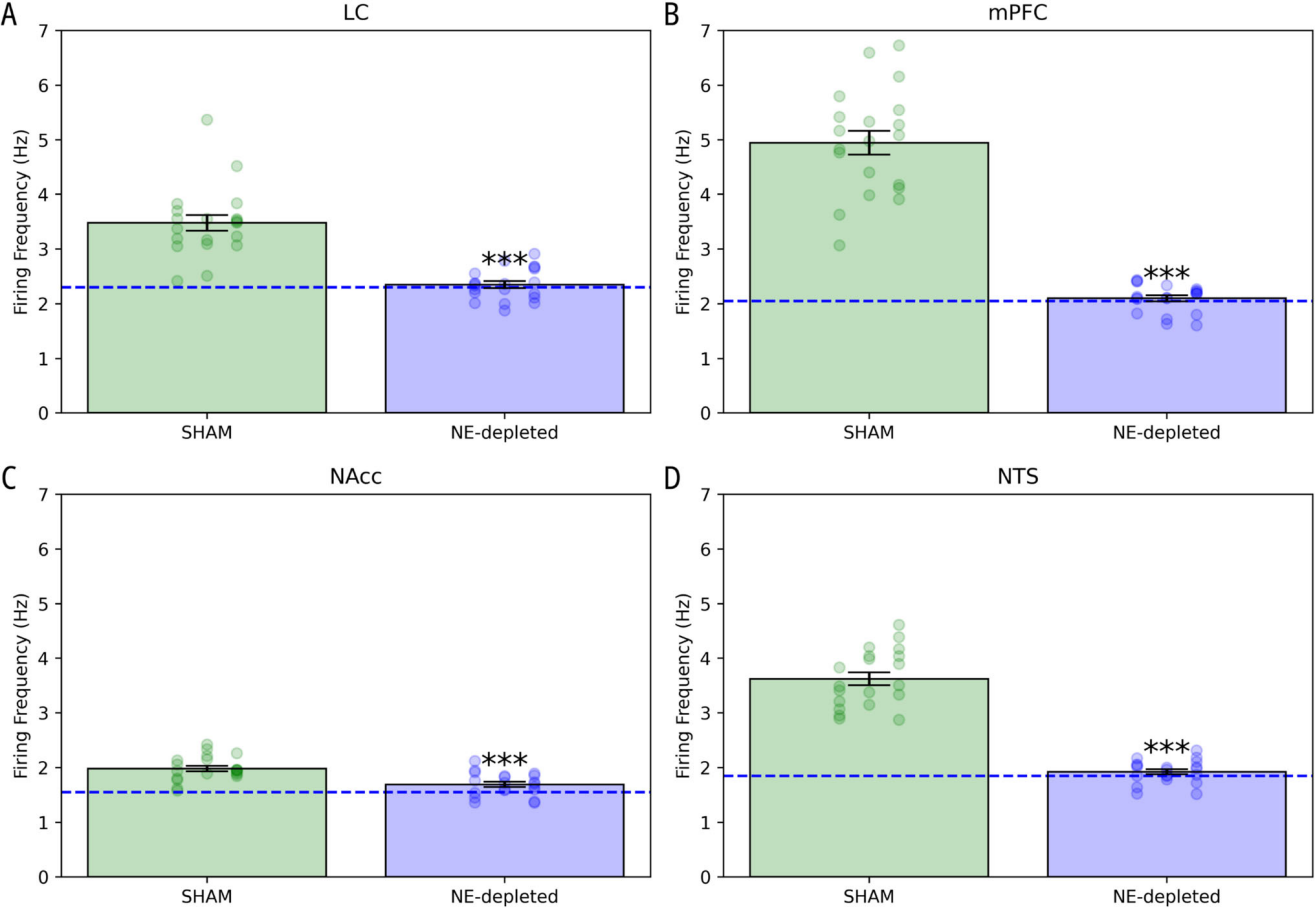
Simulation of cocaine-induced increases in baseline activity. **A** LC activity in Sham versus NE-depleted mice. **B** mPFC activity in Sham versus NE-depleted mice. **C** NAcc activity in Sham versus NE-depleted mice. **D** NTS activity in Sham versus NE-depleted mice. The dashed line represents the baseline. Data are presented as the mean values across the simulation period for each brain region (***$p < 0.001$, mean ± SEM)

The first set of results focuses on how simulated cocaine administration affects the firing activity of key brain regions in both the sham and NE-depleted conditions. The analysis examines changes in the LC, mPFC, NAcc, and NTS following cocaine injection, highlighting significant alterations in neural activity under these different conditions. A significant increase in basal activity was observed in the brain regions of the model: LC ($n=20; p<0.0001$, Mann–Whitney) (Fig. 3A), mPFC ($n=20; p<0.0001$, Mann–Whitney) (Fig. 3B), NAcc ($n=20; t(38)=4.231; p<0.0001$) (Fig. 3C), and NTS ($n=20, t(24.97)=13.37; p<0.0001$, Welch’s correction) (Fig. 3D), after administration of $20~\text {mg/kg}$ cocaine. No significant increase was observed following NE depletion in the mPFC: LC ($t(18)=0.111; n.s.$), mPFC ($t(18)=0.333; n.s.$), and NTS ($t(18)=0.585; n.s.$), except in NAcc ($t(18)=7.665; p<0.0001$).

The significant increase in basal activity across the brain regions-LC, mPFC, NAcc, and NTS-following cocaine administration can be explained by the network interactions modeled in this study. The mPFC sends excitatory signals to both the LC and NTS, enhancing their activity. In turn, the LC and NTS, both of which are key noradrenergic sources, project to the NAcc shell, contributing to the overall increase in activity. The NE projections from the LC to the mPFC further amplify this activation, creating a feedback loop that sustains heightened neural activity. These dynamic interactions likely underlie the observed elevation in basal firing rates post-cocaine administration. In contrast, NE depletion in the mPFC disrupts this excitatory signaling, resulting in no significant change in activity in most regions, except for the NAcc. This exception could stem from the direct projections from the LC and NTS to the NAcc shell, which remain active despite NE depletion in the mPFC, maintaining some level of activity in the NAcc. Thus, the model highlights how cocaine affects the entire NE circuit through complex feedback mechanisms involving these interconnected brain areas.

### Simulated NE depletion on cocaine-induced NE release in the mPFC

**Fig. 4:**
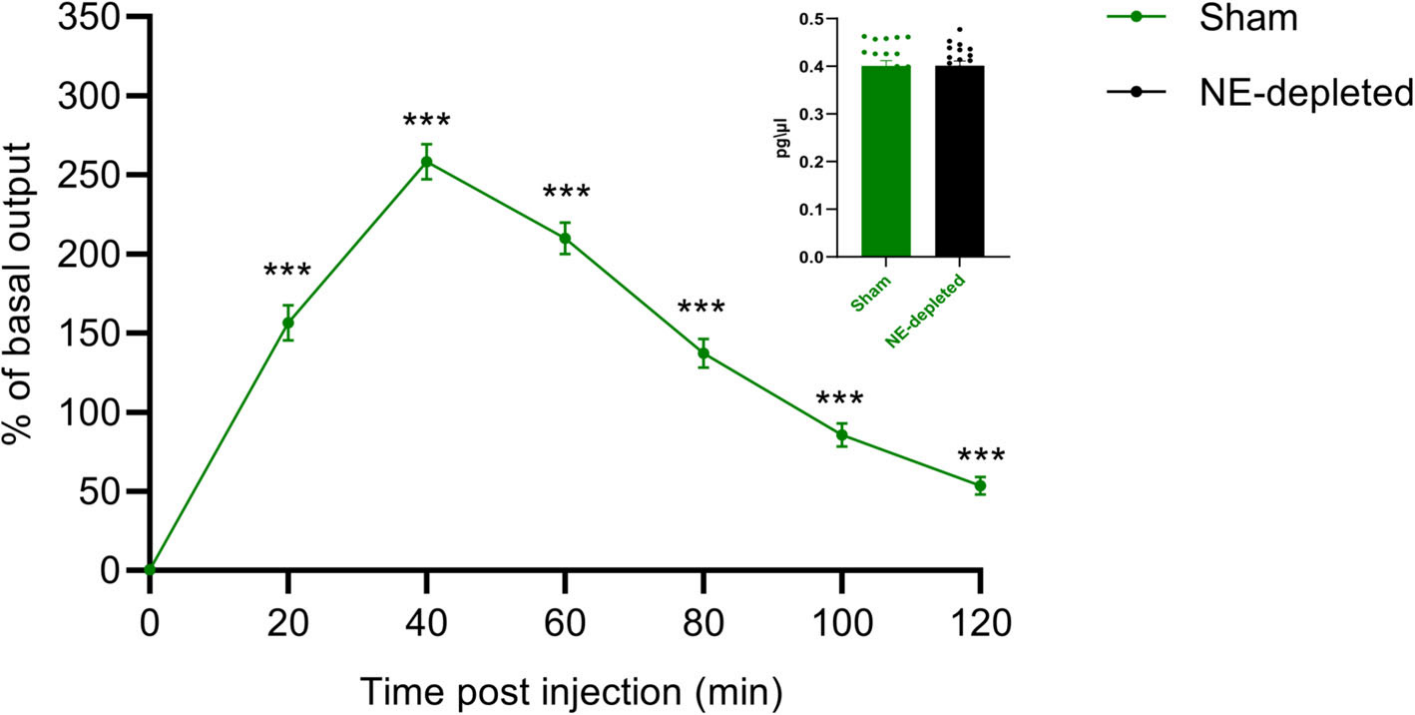
Simulation of cocaine-induced NE release in the mPFC. Cocaine administration increases NE release in the mPFC of Sham mice compared to NE-depleted mice (***$p < 0.001$, mean ± SEM). Baseline NE levels (mean ± SEM) are shown on the top right

Here, the model is used to reproduce data from an in vivo intracerebral microdialysis experiment in the mPFC, showing that NE-depleted male mice injected (i.p.) with a single dose of cocaine (20 mg/kg) exhibit specific changes in neurotransmitter dynamics. The Mann–Whitney test between groups revealed a significant increase in NE outflow in the mPFC of Sham mice at several time points (20, 40, 60, 80, 100, and 120 min) compared to NE-depleted mice (Fig. 4, left). This suggests that intact NE levels are necessary for the enhanced NE response to cocaine. Additionally, no significant difference in baseline NE release was observed between Sham and NE-depleted mice prior to cocaine administration (n=20, $t(38)=0.02$, not significant, student’s t-test) (Fig. 4, right). This indicates that the NE depletion specifically impairs cocaine-induced NE outflow rather than general baseline levels, a finding accurately replicated by the model.

### Simulated NE depletion on cocaine-induced NE release in the NAcc

**Fig. 5:**
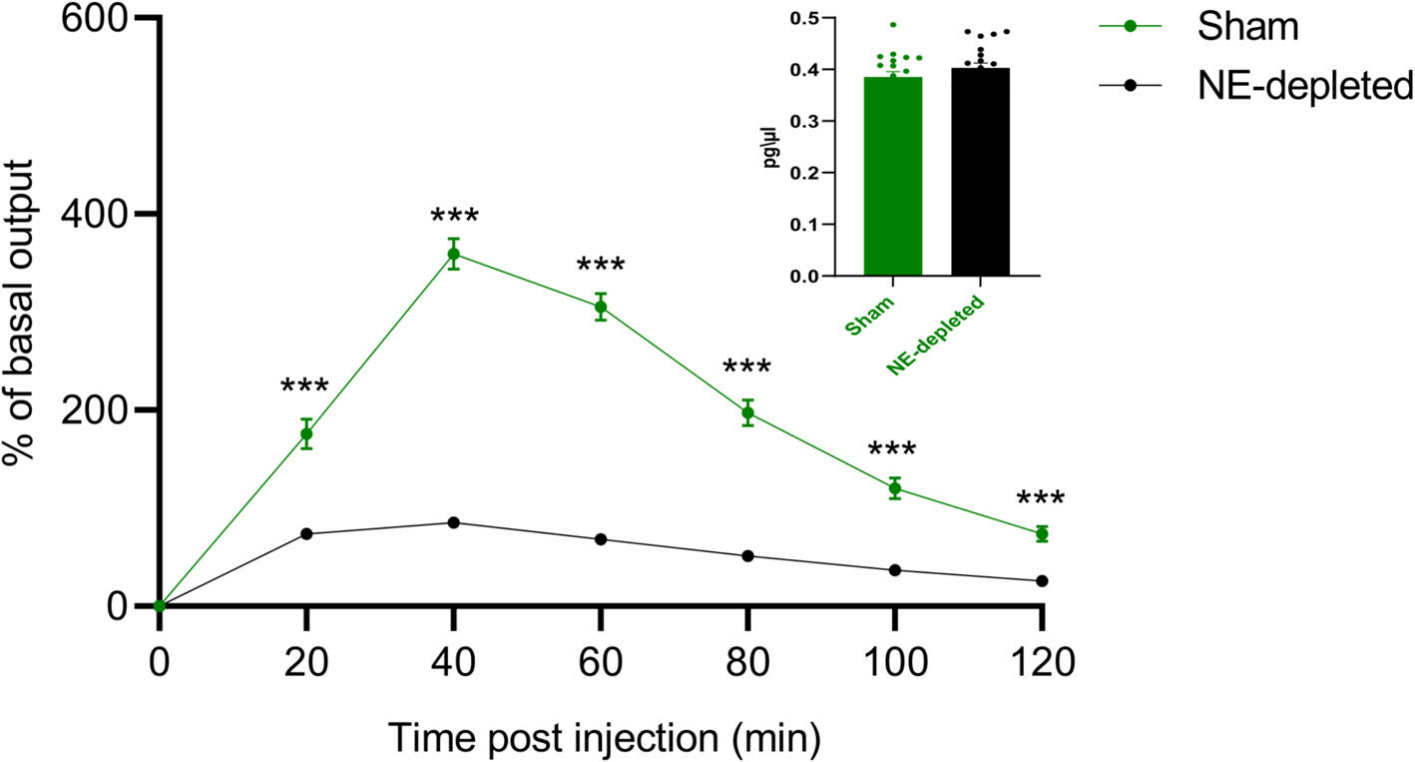
Simulation of cocaine-induced NE release in the NAcc. NE release in the NAcc, stimulated by cocaine (20 mg/kg), in Sham (green line) and NE-depleted (black line) groups (***$p < 0.001$, mean ± SEM). Baseline NE outflow in Sham and NE-depleted groups (Sham: n=10, Depleted: n=10) are shown on the top right

The simulation of NE depletion also led to a reduction in NE release in the NAcc (Fig. 5). A Mann–Whitney test indicated a significant difference in NE release between Sham and NE-depleted conditions at several time points (20, 40, 60, 80, 100, and 120 min). This suggests that NE depletion significantly reduces cocaine-induced NE release in the NAcc. Additionally, no significant differences were observed in baseline NE levels between the two conditions before cocaine administration, as indicated by the student’s t-test ($t(38)=1.23$, not significant). This confirms that the effect of NE depletion is specific to the response to cocaine, rather than altering baseline NE levels in the NAcc.

The peak observed at 40 min in the simulation likely reflects the complex dynamics of norepinephrine (NE) release in response to cocaine administration. This peak arises from the interplay between the pharmacokinetics of cocaine and the neural dynamics modulated by NE. In our model, the increase in NE release is governed by a kernel function that captures the temporal dynamics of cocaine effect on neurotransmitter levels. The function represents a gradual, time-dependent rise in NE levels, which peaks as the cocaine effect reaches its maximum, before returning to baseline. Several parameters within the model influence both the timing and magnitude of this peak. These include the rate of cocaine metabolism, the extent of NE depletion, and the parameters of the kernel function itself, such as its scaling factors. By adjusting these parameters, both the location and height of the peak can be altered. For instance, variations in the rate of cocaine metabolism can shift the onset and duration of the NE release peak, while changes to the strength of NE depletion can modulate how much cocaine affects NE levels in the NAcc.

### Model stability analysis

This section analyzes the stability of the model through a mathematical approach to the system dynamics. By defining the state vector $\textbf{y}$ to represent the system differential equations in a compact form, the analysis determines the stability conditions for both Sham and NE-depleted cases. This approach ensures the model remains mathematically robust and produces biologically realistic behavior, focusing on how stability is maintained during the transient dynamics induced by cocaine administration.

Defining the state vector $\textbf{y}$ as:

3 $$\begin{aligned} \textbf{y} = [\text {LC},\ \text {NE}\_\text {mPFC},\ \text {mPFC},\ \text {NTS},\ \text {NE}\_\text {NAcc},\ \textrm{NAcc}] \end{aligned}$$

the system can be rewritten as:

4 $$\begin{aligned} \dot{\textbf{y}}(t) = A(t)\textbf{y}(t) + \textbf{b}(t) \end{aligned}$$

hence:

5 $$\begin{aligned} {\left\{ \begin{array}{ll} \dot{y_1} = -a_{11} y_1 + a_{13} y_3 + b_1 \\ \dot{y_2} = - l k(t) a_{22} y_2 + l a_{21} y_1 \\ \dot{y_3} = - a_{33} y_3 + a_{32} y_2 + b_3 \\ \dot{y_4} = - a_{44} y_4 + a_{43} y_3 + b_4 \\ \dot{y_5} = - k(t) a_{55} y_5 + a_{51} y_1 + a_{54} y_4 \\ \dot{y_6} = - a_{66} y_6 + a_{65} y_5 + b_6 \end{array}\right. } \end{aligned}$$

where the abbreviated notation $\dot{x}$ stands for $\frac{d x}{d t}$, the state vector $\textbf{y} = [y_1,...,y_n]$, the matrix $A = [a_{ij}]$ and likewise $\textbf{b} = [b_1,...,b_n]$.

In the Sham case, all coefficients in system Eq.(4) are constant ($A(t) \equiv A, \textbf{b}(t)\equiv \textbf{b}$) since $l\equiv k(t) \equiv 1$. If *A* is non-singular there is therefore a unique equilibrium point $\bar{{\textbf {y}}} = A^{-1} {\textbf {b}}$ which is asymptotically stable if and only if all eigenvalues of *A* have negative real part: $\forall \lambda \in \sigma (A): \mathbb {R}(\lambda ) < 0$ (Brugnano and Trigiante 1996; Cross 1978; Lakshmikantham and Trigiante 2002; Poole 2015). When fitting the system parameters it is appropriate to prefer combinations that allow for an asymptotically stable solution, since this condition matches reality. Furthermore, using initial values that are arbitrarily close to the asymptotically stable solution of each system and using an appropriate integration method ensures safety from numerical instabilities or artifacts.

The depletion is modeled by reducing the reactivity of $y_2$ by means of scaling its derivative by a factor *l*. The system with applied depletion becomes:

6 $$\begin{aligned} \dot{\textbf{y}} = A_l \textbf{y} + \textbf{b} \end{aligned}$$

where $$A_l = A$$ except for the two elements $$a_{l21} = l a_{21}$$ and $$ a_{l22} = l a_{22}$$. System Eq.(6) is still a constant coefficients linear system, hence the same equilibrium and stability conditions discussed for Eq.(4) apply.

The cocaine effect is modeled in Sham and depleted systems by reducing the NE damping factor in a time dependent manner. In particular the system becomes:

7 $$\begin{aligned} \dot{\textbf{y}} = A_c(t) \textbf{y} + \textbf{b} \end{aligned}$$

where $A_c(t) \equiv A$ except for the two elements $a_{c22} = k(t) a_{22}$ and $a_{c55} = k(t) a_{55}$; *k*(*t*) is defined in Eq.(2). We have that $k(t) \in (0,1]$, $k(t) \equiv 1$ for $t < t_c$ and $\lim _{t\rightarrow \infty } k(t) = 1$; since the system is asymptotically stable when $k(t) = 1$, we can assume that the system remains asymptotically stable regardless of the transient dynamic phase starting at $t_c$.

## Discussion

Over the years, research has primarily focused on the role of dopamine (DA) in the rewarding process. However, recent studies emphasize the critical involvement of the norepinephrine (NE) system in the reinforcement effects of various classes of drugs (for a review, see Downs and McElligott (2022)). To investigate this system, a new differential equations model is introduced (Caligiore et al 2021) to capture the key features of complex NE circuits (Wydra et al 2013; Ahmadian-Moghadam et al 2018; Ly and Root 2021). The focus is on NE circuits to better understand how the medial prefrontal cortex (mPFC) modulates cocaine-induced NE release in the nucleus accumbens (NAcc). First, the model replicates cocaine-induced NE release in the mPFC (Florin et al 1994; Ventura et al 2007; Devoto et al 2014) and simulates the abolition of this increase through selective prefrontal NE depletion, as reported previously (Ventura et al 2007).

The simulations show that the model reproduces cocaine-induced NE increases in the mPFC, but, unlike in real mice, only a minimal increase in NE occurs without functional relevance (Fig. 4). This discrepancy may arise from the exclusion of other neurotransmitters in the model. Additionally, the model predicts cocaine-induced NE release in the NAcc (Fig. 5). Previous studies demonstrate that psychostimulants elicit NE release in the NAcc (McKittrick and Abercrombie 2007), but no data exist on accumbal NE release following prefrontal NE depletion. The model further suggests that prefrontal NE depletion leads to a reduction in accumbal NE outflow after cocaine administration (Fig. 5).

The literature reports that the mPFC exerts an excitatory effect on the NTS (Owens et al 1999). Additionally, Schmidt et al (2017) found that cocaine increases extracellular NE by blocking its plasma membrane transporter (NET), which in turn activates mPFC glutamatergic neurons. Consistent with this, the model results suggest that, in the absence of mPFC-mediated excitation of the NTS, a significant reduction in NE release in the NAcc would occur. This finding introduces, for the first time, a complex circuit in which the mPFC regulates NE release in the NAcc through excitatory control of the NTS (Carter 1982; Geisler and Wise 2008).

In addition to NE release, the model suggests changes in neuronal activity in brain regions examined after cocaine administration in both Sham and NE-depleted mice (Fig. 3). In particular, the prefrontal firing rate increases after cocaine administration, as previously reported by Koulchitsky et al (2012), but this effect is abolished by prefrontal NE depletion. This effect can be explained by considering the role of prefrontal NE transmission on glutamatergic neuron activity (Schmidt et al 2017). In NE-depleted mice, the lack of NE release could reduce (or prevent) the increased firing rates of these neurons. Moreover, cocaine increases the firing rate in both LC and NTS, with this increase being reduced (or abolished) in NE-depleted mice. Excitatory projections from mPFC to NTS and LC have been reported (Jodo et al 1998; Eden and Buijs 2000; Owens et al 1999), and, as discussed previously, NE depletion could prevent glutamatergic activation, suggesting a critical role for prefrontal NE on NTS and LC projections and activity.

The last brain region investigated by the model is the NAcc, which, as previously reported, shows increased activity following cocaine injection (Carelli 2000). This activity is also evident in NE-depleted animals. This result could be related to the continuous release of NE in the model after depletion, or it could be influenced by other neurotransmitters not considered in the model. Future in vivo investigations using electrophysiology could address this question.

The stability analysis demonstrates that the model reliably reproduces the results using solid mathematical formulations, with stability conditions confirmed for all virtual mice. This is a key factor in validating the model effectiveness (Fornari et al 2020; Shi et al 2020). By proposing a potential mechanism underlying the role of the prefrontal cortex in cocaine-induced noradrenaline release, and supporting it with stability analysis, the model gains additional credibility. The stability suggests that the mechanism is likely valid, rather than being an artifact of parameter tuning. This reinforces the idea that the model predictions are grounded in meaningful system dynamics, rather than arbitrary adjustments.

Overall, these findings carry significant implications for understanding the neural circuits involved in reinforcement and addiction. The alterations in firing rates and NE release dynamics not only shed light on the neuropharmacological effects of cocaine but also provide insights into potential therapeutic targets for addiction treatment. Furthermore, the observed interactions between mPFC, NTS, and NAcc highlight the importance of integrated circuit dynamics, which could lead to novel hypotheses regarding individual variability in response to psychostimulants. The stability analysis reinforces the robustness of these findings, indicating that the model’s predictions hold across various parameter settings, further supporting their biological relevance.

In light of the findings from this study, it is also worthwhile to consider how these results relate to computational models of neuromodulation, particularly in the context of reinforcement learning and decision-making. Models of neuromodulation, such as those proposed by Angela and Dayan (2005), suggest that neuromodulators like norepinephrine (NE) play a critical role in regulating attention and the updating of cognitive states based on uncertainty. The observed changes in NE release in response to cocaine administration in our model align with the concept that NE serves as a modulator that adjusts the salience of information, thereby affecting the learning process and behavioral output in a dynamic environment. In particular, the differential NE dynamics observed in the mPFC and NAcc during cocaine exposure could be viewed as reflecting an alteration in the system’s capacity to encode or prioritize new information under altered motivational states, an idea consistent with the framework of neuromodulation proposed by Angela and Dayan (2005). Moreover, Dayan (2012) presents a comprehensive overview of computational neuromodulation, highlighting the importance of neuromodulatory systems in shaping learning processes. Dayan emphasizes the role of neuromodulators in modulating learning rates, attention, and the updating of action values, which could be instrumental in understanding how the brain integrates new experiences and modifies its internal models of the world. The present study, by focusing on the interaction between NE in the mPFC and NAcc, provides insights into how cocaine-induced changes in these systems may affect the overall learning process, potentially leading to maladaptive behaviors characteristic of addiction. These computational perspectives offer a valuable framework for interpreting the dynamic neural interactions captured in our model and provide a foundation for future research into how disturbances in neuromodulatory systems may contribute to pathological behaviors.

In addition to the theoretical insights provided by our computational model, future experimental studies could play a key role in validating and expanding upon the framework presented here. In vivo experiments, such as electrophysiological recordings from brain regions like the mPFC, NAcc, and LC during cocaine administration, could directly assess the model’s predictions regarding firing activity and neurotransmitter release. For example, chronic or acute pharmacological manipulations of NE or other neuromodulators could help determine the causal relationships between these systems and their contribution to the reinforcement process. Additionally, optogenetic techniques could be used to selectively modulate neuronal activity in specific brain regions, providing a means to test the functional relevance of different pathways in the model, such as the excitatory control of NTS by mPFC.

Furthermore, the proposed framework could be applied to investigate the effects of other neurotransmitters, such as dopamine and serotonin, on the dynamics of the neural circuits involved in addiction. Behavioral assays, combined with pharmacological and optogenetic manipulations, could help to probe the link between neural activity and behavioral outcomes. Such experimental efforts would not only validate the model but also provide new insights into the broader mechanisms of neuromodulation and their role in cognitive functions and disorders, including addiction. By integrating computational models with experimental neuroscience, we can deepen our understanding of the brain’s dynamic response to external stimuli and potentially uncover novel therapeutic targets for psychiatric conditions.

## Conclusions

Understanding cocaine mechanisms of action is highly complex due to the involvement of multiple factors (Bravo et al 2022). This study utilizes computational modeling to explore the role of NE transmission in greater depth. After successfully replicating NE release in the mPFC of both control and NE-depleted mice, the model was used to predict NE release in the NAcc under the same conditions. Despite its limitations-such as focusing on only four brain regions, excluding other neurotransmitter systems, and simplifying cocaine effects on the brain-the results offer valuable insights into the NE pathways through which cocaine exerts its influence. Future efforts should aim to refine the model by incorporating interactions with other neurotransmitters and additional brain regions. In vivo experiments targeting selective modulation of the NTS would also be essential for validating and expanding these findings.

## Data Availability

https://github.com/ctnlab/NTS_NE/
